# Conformational Sampling in Template-Free Protein Loop Structure Modeling: An Overview

**DOI:** 10.5936/csbj.201302003

**Published:** 2013-02-25

**Authors:** Yaohang Li

**Affiliations:** a Department of Computer Science, Old Dominion University, Norfolk, VA 23529, USA

## Abstract

Accurately modeling protein loops is an important step to predict three-dimensional structures as well as to understand functions of many proteins. Because of their high flexibility, modeling the three-dimensional structures of loops is difficult and is usually treated as a “mini protein folding problem” under geometric constraints. In the past decade, there has been remarkable progress in template-free loop structure modeling due to advances of computational methods as well as stably increasing number of known structures available in PDB. This mini review provides an overview on the recent computational approaches for loop structure modeling. In particular, we focus on the approaches of sampling loop conformation space, which is a critical step to obtain high resolution models in template-free methods. We review the potential energy functions for loop modeling, loop buildup mechanisms to satisfy geometric constraints, and loop conformation sampling algorithms. The recent loop modeling results are also summarized.

## Introduction

A loop, also called a coil, is a flexible segment of contiguous polypeptide chain that connects two secondary structure elements in a protein. The loop regions play critical roles in protein functions, such as involving in catalytic sites of enzymes [1], contributing to molecular recognition [2–4], and participating in ligand binding sites [5–7]. As a result, accurate prediction of the loop regions conformations in proteins is important for a variety of structural biology applications, including determining the surface loop regions in comparative modeling [8], defining segments in NMR spectroscopy experiments [9], designing antibodies [10], identifying function-associated motifs [11], and modeling the dynamics of ion channels [12, 13].

**Figure 1:**
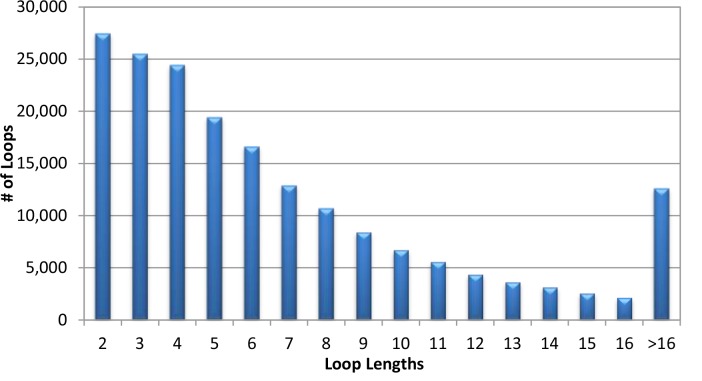
Distribution of loop lengths in the protein chain list generated by the PISCES server [21] on Aug. 28, 2012 containing 13255 chains with 2.0A resolution, 90% sequence identity, and 0.25 R-factor cutoff.

**Figure 2:**
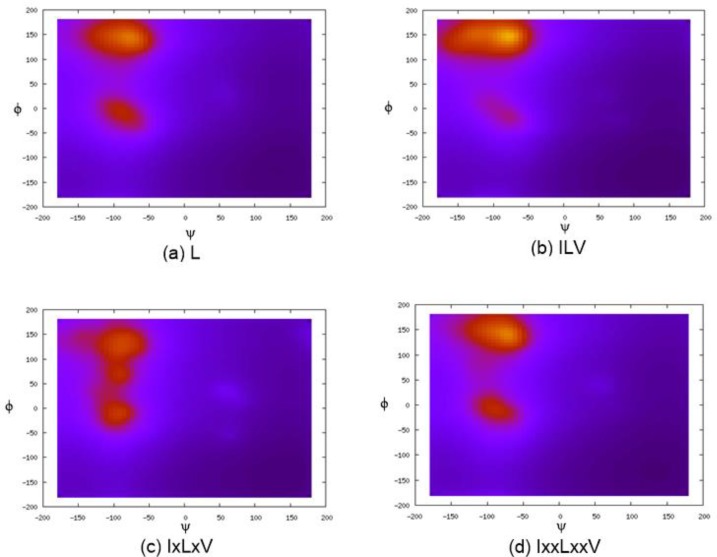
*φ*-*ψ* propensity maps of Leucine in the loops in presence of hydrophobic neighbors (ILE and VAL): (a) LEU as a singlet; (b, c, d) LEU with ILE and VAL as the nearest, one position away, and two positions away neighbors in sequence. The nearest and second nearest neighbors have strong influences to the backbone torsion angle conformations of Leucine and the influences from further neighbors are significantly weakened.

According to the loop length distribution illustrated in Figure 1, 93.2% of loops have lengths ranging from 2 to 16 residues, although sometimes loops can stretch much longer. Nevertheless, due to their high flexibility, loops regions are usually more difficult to model and analyze than the other secondary structures such as helices or strands. Indeed, in many (complete) protein models derived from computational methods, the loop regions, particularly the long ones, are the places contributing a lot of error [77]. At the early attempt of loop modeling, Flory [14] assumed that the backbone torsion angles corresponding to one residue are random, more precisely, statistically independent from the backbone torsions of its neighbors. However, more and more experimental [15], evolutional [16], and statistical [17] data have shown that loops are far from random and the nearby residue neighbors in sequence are sufficiently strong to account for substantial changes in the overall structure of loops. Figure 2 shows the *φ*-*ψ* propensity maps of Leucine in loops when the hydrophobic residues (ILE and VAL) are presented as neighbors at different distances. One can find that the backbone dihedral angle conformations of Leucine have strong correlation with the types of residues at the nearest and second nearest neighboring positions. However, such influences from residues at further positions are much weaker. The *φ*-*ψ* propensity maps of Leucine with ILE and VAL as two positions away neighbors are almost indistinguishable to the one of singlet Leucine, indicating that influences from neighboring loop residues two positions or further away are negligible. Moreover, studies have demonstrated that the identical peptide segments can adopt completely different structures in different proteins [18, 19]. Hence, in addition to the residues in a loop, the residues surrounding the loop structure are also important to determine its conformation, particularly for a loop deeply embedded in the protein structure. Furthermore, the distance between the anchor points in the rest of the protein that spans the loop likely influences the loop conformation as well, particularly when the loop is short. To facilitate studies on 3D structures of loops, the Protein Coil Library [20] maintains the structures of all loop segments derived from protein structures presented in Protein Data Banks (PDB).

In general, loop structure modeling methods can be categorized into template-based (database search) methods and template-free (*ab initio*) methods. The template-based methods [22–25] search PDB for loop structure templates that fit the geometric and topologic constraints of the loop stems. The template-based methods highly depend on the quality and number of known structures in the PDB. Due to the fact that the number of possible loop conformations grows exponentially with lengths, the template-based methods are limited to relatively short loops. In contrast, the template-free methods can avoid this problem by sampling loop conformation space guided by energy functions. In this mini-review, we focus on the template-free methods only.

The template-free loop modeling problem is regarded as a “mini protein folding problem” [47] under geometric constraints, such as loop closure and avoidance of steric clashes with the remainder of the protein structure. Similar to the protein folding problem, modeling steps including coarse-grained sampling, filtering, clustering, fine-grained refining, and ranking are often found in most loop structure prediction methods. During the coarse-grained sampling step, guided by knowledge- or physics-based energy functions, the loop conformation space is explored to produce a large ensemble of reasonable, coarse-grained models satisfying geometric constraints. These coarse-grained models usually use reduced representations for loop structures, such as *φ*-*ψ* angles, backbone atoms, Cα atoms only [59], or side chain centers of mass [68]. Afterward, the coarse-grained models are filtered to eliminate the unreasonable ones in the ensemble [60] and then the representative models are selected by a clustering algorithm to reduce redundancy. These representative models are used to build fine-grained models in the refining phase, usually guided by a more accurate energy function associated with more structural information such as side chains and hydrogen atoms. Finally, in the ranking phase, the final models are assessed and the top-ranked ones are determined as the predicted results [62]. Among all these modeling steps, the coarse-grained sampling phase is of particular importance – if the sampling process cannot reach conformations close enough to the native, it is unlikely to obtain a high-resolution near-native model eventually. Moreover, the success of sampling relies on the underlying energy (scoring) functions, which are required to provide not only accurate, but also sensitive guidance to the sampling process to explore the protein loop conformation space.

There has been a lot of work done in modeling proteins loops since late 1960s. Limited by length, it is not our intention to provide a thorough review of loop modeling approaches in this mini review. Instead, we focus on the recent computational sampling approaches developed for protein loop structure prediction using template-free methods. We put our emphasis on the important factors that impact loop conformation sampling efficiency, including energy functions for modeling loops, loop buildup algorithms to satisfy geometric constraints, and coarse-grained sampling algorithms. The results of recent works in loop structure prediction are also summarized.

## Energy Functions for Loop Modeling

According to Anfinsen's thermodynamics hypothesis [26], the native protein structure having the native structure conformation has the minimum Gibbs free energy of all accessible conformations. Similar to the general protein folding problem, many efforts of loop modeling focus on minimizing the protein potential energy described by physics-based energy functions. Zhang et al. [27] designed a simplified soft-sphere potential to fast construct loops. Cui et al. [28] developed a grid-based force field for their Monte Carlo (MC) sampling approach. More recent works take advantage of the existing force fields and solvent models popularly used in molecular simulation. Rapp and Friesner [29] and de Bakker et al. [30] used the AMBER [31] force field with a Generalized Born solvent model. Spassov et al. [32] adopted the CHARMM [33] force field in their LOOPER algorithm. The Protein Local Optimization Program (PLOP) [34] developed by Jacobson et al. is based on the OPLS-AA [35] force field with Surface Generalized Born (SGB) solvent model. Rapp et al. [36] also used OPLS-AA/SGB to reproduce loop geometries in experimental solution structures. Zhu et al. [37] included a hydrophobic term in SGB solvation model and achieved accuracy improvement in long loops ranging from 11 to 13 residues. Felts et al. [38] incorporated Analytical Generalized Born plus Non-Polar (AGBNP) [39] implicit solvent model into PLOP. Sellers et al. [40] used MM/GBSA (Molecular Mechanics/Generalized Born Surface Area) energy [41] for loop refinement in comparative modeling. Danielson and Lill [80] also used MM/GBSA to study flexible loops interacting with ligands. Fogolari and Tosatto [42] took advantage of the concept of “colony energy” by considering the loop entropy, an important component in flexible loops, as part of the total free energy.

Since the main goal in loop structure prediction is to model loop conformation with high accuracy instead describing the underlying physics [47], an alternative approach to assess the correctness of a loop conformation is knowledge-based energy functions. The rationale of knowledge-based energy function is to obtain “pseudo energy” based on statistical preferences of conformations for different geometries as obtained from the database of known protein structures. Compared to the physics-based energy functions, the knowledge-based energy functions have several attractive advantages. First of all, the knowledge-based energy functions implicitly capture interactions that are difficult to model in physics-based energy functions. Secondly, the knowledge-based scoring functions usually do not require all atom information of the loops, which is ideal to rapidly generate coarse-grained models. Thirdly, the knowledge-based potentials tend to be “softer” to tolerate structural imperfection – allowing better handling of uncertainties and deficiencies of the computer generated models.

Sippl's potentials of mean force approach [43] is one of the most notable methods to obtain knowledge-based energy functions. According to the inverse-Boltzmann theorem, the knowledge-based energy potential *U(f)* for a feature *f* is calculated as

$$u(f)=-kTln\frac{P_{obs}(f)}{P_{ref}(f)},$$

where *k* is the Boltzmann constant, *T* is the temperature, *P*_obs_(ƒ) is the observed probability in the database of known structures, and *P*_*ref*_ *(ƒ)* is the probability of the reference state. Possible features to which a pseudo-energy term can be assigned include pairwise atom distances, torsion angles, amino acid contacts, side chain orientation, solvent exposure, or hydrogen bond geometry. For example, DFIRE [44] and DOPE [45] energy functions are built on the statistics of distance of pair-wise atoms. The dipolar DFIRE (dFIRE) [46] adds orientation-dependent terms to DFIRE by treating each polar atom as a dipole. Rata et al. [17] developed a statistical potential for loops based on adjacent *φ*-*ψ* pair distribution in the context of all possible combinations of local residue types. Liang et al. (OSCAR-loop) [64] optimized the knowledge-based potential for backbone torsion angles as Fourier series. Galaktionov et al. [65] designed penalty functions based on residue-residue contact map representations to model loops over 20 residues. Burke and Deane [69] calculated a sequence-based scoring function to estimate the compatibility of a sequence with a certain loop class.

In practice, physics- and knowledge-based energy terms are also often combined together to enhance the accuracy of the energy functions for loop prediction. Fiser et al. [47] used an energy function where stereochemical features are obtained from CHARMM-22 [33] force field while the non-bonded interactions, solvation, torsion angle preferences are derived from statistics. This energy function and the corresponding loop modeling method are adopted in the Modeller program. Rohl et al. [48] and later Mandell et al. [49] used the Rosetta scoring function [50], a hybrid scoring function which has demonstrated its effectiveness in CASP experiments. Xiang et al. [51] developed a combined energy function with force-field energy and RMSD (Root Mean Square Deviation) dependent terms, which is used in their LOOPY program.

**Figure 3:**
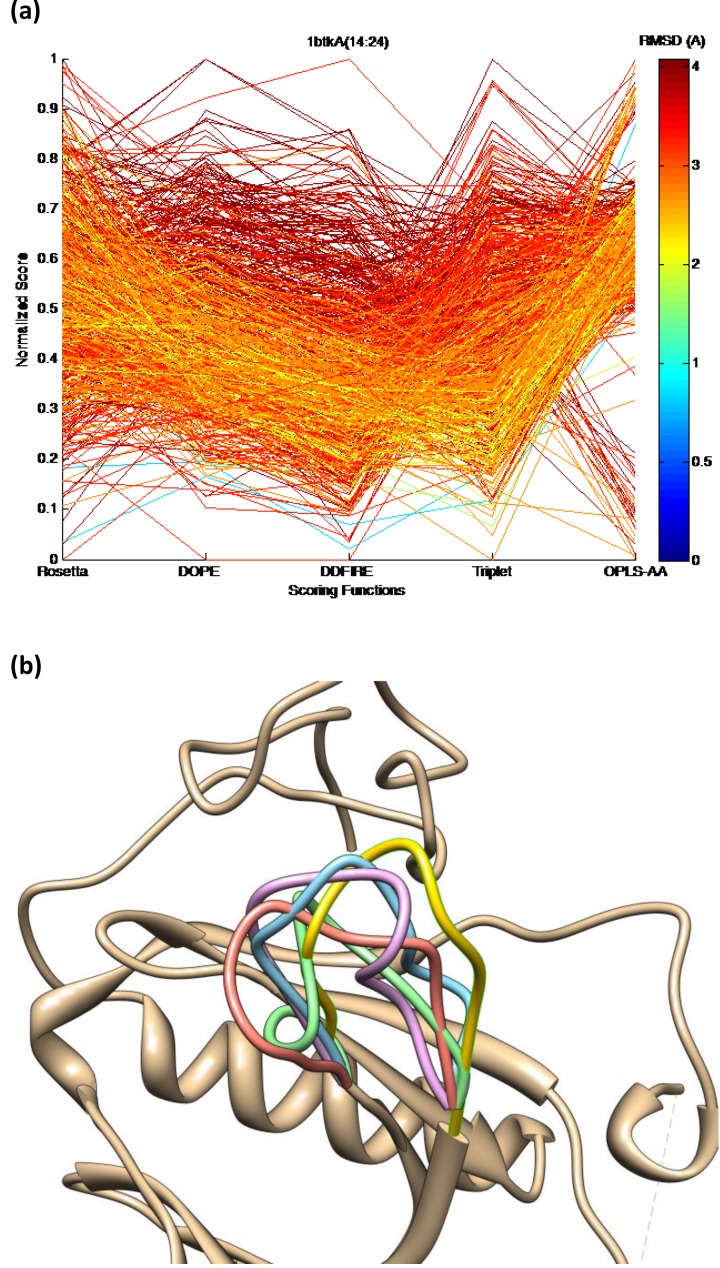
**(a)** Multiple energy functions coordinate plot of loop 1btkA(14:24) decoys in Jacobson loop decoy set using 5 energy functions, including Rosetta, DOPE, dDFIRE, backbone torsion potential using triplets, and OPLS-AA. All scores are linearly normalized in [0, 1]. RMSD is calculated for all backbone atoms in the loop. None of these energy functions can identify a near native decoy (< 1.0A) with the lowest energy value. **(b)** Native loop (gold) and loop decoys with lowest scores in Rosetta (blue, 2.73A), DOPE and dDFIRE (green, 2.85A), Triplet (red, 2.34A), and OPLS-AA (purple 2.27A).

Although quite a few energy functions derived by different manners are available for loop structure modeling, currently there does not exist a superiorly accurate energy function that can always differentiate the near native structures from the other incorrect ones in all protein loops. Figure 3(a) depicts a coordinate plot of multiple energy functions on decoys of 1btkA(14:24) contained in Jacobson loop decoy set [63] using a variety of physics-based, knowledge-based, or hybrid energy functions, including OPLS-AA [35], Rosetta [50], DOPE [45], dDFIRE [46], and backbone torsion potential using triplets [17]. None of these energy functions can correctly identify a near native decoy (< 1.0A) with the lowest energy value, although some near-native decoys exhibit low energy values in various energy functions. Figure 3(b) shows the loop decoy structures with the lowest energy values in different energy functions.

## Loop Closure

**Figure 4:**
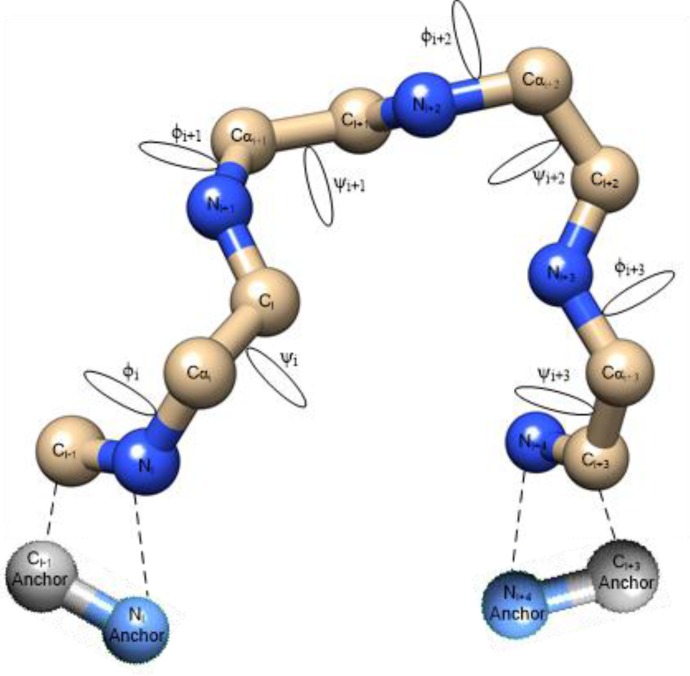
Addressing *φ*-*ψ* angles of a 4-residue loop to bridge the gap between the targeted anchored points

The computer-generated loop models during the sampling process must satisfy the loop closure condition, i.e., the endpoints (C- and N-terminals) of a loop model must seamlessly bridge the anchored endpoints (C- and N-anchors) of the given protein structure. Figure 4 depicts the loop closure problem.

Computational methods enforcing loop models to satisfy loop closure constraints include energy penalty [52], finding analytical solutions [49, 53, 54], random tweak [55], wriggling [56], Cyclic Coordinate Descent (CCD) [57], or bi-directional inverse kinematics [58]. The energy penalty approach adds an additional term to the energy function to penalize deviations between the loop endpoints and the target anchor points [52]. The original method for obtaining analytical solutions is described in the pioneer work by Go and Scheraga [78]. Wedemeyer and Scheraga [53] derived the analytical solutions by determining the real roots of a polynomial, which lead to the solutions for closure of 6 backbone torsion angles in tripeptides. Coutsias et al. [54] and Mandell et al. [49] generalized the applicability of the analytical solutions to 6 not necessarily consecutive torsion angles in peptides of any length while small perturbations in bond angles and peptide torsion angles are also allowed. The random tweak method [55] is carried out by applying small random changes to *φ*-*ψ* angles and then using an iterated linearized Lagrange multiplier algorithm to satisfy the loop closure constraints with minimal conformational perturbations. Wriggling [56] takes advantage of the linear dependency of every four angles of rotation to keep the combined motion of loop localized. The CCD algorithm [57] treats the loop closure problem as an inverse kinematics problem, which fixes one loop endpoint at the one anchor and then iteratively modifies the *φ*-*ψ* angles in sequential order to minimize the distance between the other loop endpoint and the target anchor. The Full CCD (FCCD) algorithm [59] extends the applicability of CCD to a reduced loop representation with Cα atoms only by using a singular value decomposition-based optimization of a general rotation matrix. The bi-directional inverse kinematics method [58] adopts the “meet in the middle” strategy by generating half-loops from both C- and N-anchors and then assembles the endpoints of the half loops, which is particularly suitable for modeling long loops. In [60], Soto et al. provided a comparison of effectiveness and computational performance among various loop closure algorithms.

The above methods ensure loop closure, however, without considering the other geometric constraints such as steric clashes. Several methods have been proposed to account for additional geometric constraints in loop modeling. Xiang et al. [51] imposed a non-bonded energy term on the iterated Lagrange multiplier in the random tweak method to avoid steric clashes while satisfying loop closure simultaneously. Liu et al. [61] designed a self-organizing algorithm by performing fast weighted superimpositions of rigid fragments and adjusting distances between random atom pairs to resolve steric clashes, where not only loop closure, but also steric, planar, chiral, and even constraints derived from experiments can be satisfied simultaneously.

## Loop Conformation Sampling

The loop conformation sampling is usually done by sampling backbone torsion angle conformations by deterministic or statistical sampling methods. In practice, it is not computationally feasible to sample all combinations of discretized torsion angles for a relatively long loop. Indeed, a large portion of these torsion angle combinations are infeasible due to steric clashes, unable to close, excluded volume for side chains, etc. In principle, both deterministic and statistical sampling techniques try to avoid these infeasible conformations as many as possible.

Deterministic sampling intends to find all possible loop conformations with reasonable but diversified structures. Galaktionov et al. [65] built loops (up to 12 residues) based on all possible combinations of local minima of empirical conformational energy for *φ*-*ψ* angles of each residue. Jacobson et al. [34] and Zhu et al. [37] developed rotamer libraries in PLOP for backbone torsion angles from high-resolution protein structure database. Then, loops are built up from the rotamer libraries while a variety of screening criteria, including effective resolution, clashes, impossible closure, deviation from protein body, and space for side chains, are used during sampling to eliminate as many infeasible structures as possible. Zhao et al. [79] extended the rotamer libraries to dipeptide segments to model long loops over 13 residues. Spassov et al. [32] performed a systematic search of *φ*-*ψ* angles belonging to one of the low energy basins in the iso-energy contour of local interactions.

Instead of attempting to generate all reasonable loop conformations, statistical sampling methods focus on the statistical favorability of conformations likely yielding low energy in the energy landscape. In Modeller [47] and LOOPY [51], a lot of random loop conformations are generated and then optimized by energy minimization. In RAPPER [66], an ensemble of conformations with pair-wise RMSD greater than 0.2A is collected using a round-robin algorithm, in which a suitable *φ*-*ψ* combination satisfying geometric constraints is selected to gradually grow the loops. Lee et al. [75] produced loop conformations by sequentially adding randomly chosen 7-residue fragments obtained from known structure database. Ring and Cohen [70] sampled loop conformations with Genetic Algorithms (GA). More popularly, the Markov Chain Monte Carlo (MCMC) method [27, 28, 48, 49, 52, 61, 63, 64, 67, 68] is adopted to explore loop conformation space. The fundamental idea of MCMC is to perform local MC moves to propose new loop conformations satisfying loop closure and other geometric constraints without disturbing the rest of the protein structures and then decide the acceptance according to Metropolis acceptance-rejection criterion [71]. Various techniques have been used to enhance MC sampling efficiency, including simulated annealing [27, 28, 48, 49, 52, 64], hierarchical MC [63], replica exchange [67], and configuration-biased MC [68].

Generally, from algorithm point of view, GA is usually more effective than MC in terms of number of iteration steps to convergence, mainly due to better local minima escaping capability in GA when genetic operators such as crossover are employed [86]. However, in loop modeling, new conformations generated by crossover or mutation likely break the loop closure condition while potentially cause steric clashes. Additional quality control steps, potentially computationally costly, are necessary to correct these violations in geometric constraints [86]. In contrast, local MC moves in MCMC sampling guarantee satisfaction in geometric constraints and thus is more favorable in exploring loop conformation space.

After sampling, a set of coarse-grained loop models exhibiting good geometric properties are generated. Refining loop models, usually guided by a more accurate and sensitive energy potential associated with more structural information such as side chain and hydrogen atoms, is needed to build fine-grained models. Similar to refining the complete protein structure, commonly used approaches to refine loop structures include local optimization [34], MC [81], and more often Molecular Dynamics (MD) simulations [82–85]. Furthermore, it is important to notice that coarse-grained and fine-grained sampling can be combined together to enhance exploration of loop conformation space, as an example shown in [67] where MC and MD simulations are integrated by a replica exchange algorithm.

Each loop modeling method has certain inevitable inaccuracy due to the limitation of sampling methods, uncertainty in energy functions, numerical errors, etc. A new strategy is to integrate different modeling methods to account for different sources of inaccuracy. Deane and Blundell [76] generated consensus predictions from two separate algorithms based on real fragments and computer generated fragments, respectively. Li et al. [72] developed a Pareto Optimal Sampling (POS) method based on the Multi-Objective Markov Chain Monte Carlo (MOMCMC) algorithm [73] to sample the function space of multiple knowledge- and physics-based energy functions to discover an ensemble of diversified structures yielding Pareto optimality. Jamroz and Kolinski [74] proposed a multi-method approach using MODELLER, Rosetta, and a coarse-grained de novo modelling tool, which leads to better loop models than those generated by each individual method.

## Recent Loop Prediction Results

Table 1 summarizes the energy functions, sampling methods, and loop closure mechanisms and Table 2 lists the loop prediction accuracies in recently (since 2000) published works. Due to advances in computational loop modeling methods, highly accurate models with resolution comparable to experimental results have been achieved in quite a few methods shown in Table 2 for loops less than 8 residues. Several recent methods [37, 49, 72] can predict loop conformations within or close to 1A RMSD for loop targets up to 13 residues. Another important factor leading to loop modeling improvement is the stable growth of the number of known structures in PDB, which allows one to derive more sensitive knowledge-based energy functions, calibrate physics-based energy functions to achieve higher accuracy, and obtain richer loop fragments or rotamer libraries. Nevertheless, for the very long loops, for example, those over 18 residues, significant breakthrough has not been reported yet. According to Galaktionov et al. [65], modeling very long loops is a “different problem” due to their significantly higher flexibility compared to relatively short loops, which demands “different methodological approaches.”

**Table 1:** Energy functions, sampling methods, and loop closure mechanisms in recently published loop structure modeling works.

Loop Modeling Methods	Energy Functions	Coarse-grained Sampling	Fine-grained Sampling	Loop Closure
Fiser et al. [47] (2000) (Modeller)	Statistical potential integrating simple restraints or pseudo-energy terms	Random Buildup	Conjugate gradients – MD with simulated annealing – Conjugate gradients	Guaranteed in random buildup
Deane and Blundell [88] (2000) (PETRA)	Statistical all-atom, distance-dependent conditional probability function	Search Polypeptide Fragment Database	-	Filtering based on closure gap
Deane and Blundell [76] (2001) (CODA)
Xiang et al. [51] (2002) (LOOPY)	Colony energy	Random Buildup	Fast torsion minimizer	Random tweak
DePristo et al. [66] (2003)	RAPDF-1 and RAPDF-2 (coarse)	Sample dihedral angles from fine-grained torsion angle state sets	Limited-memory BFGS	Gap-closure restraint
De Bakker et al. [30] (2003) (RAPPER)	AMBER-GBSA (fine)
Rohl et al. [48] (Rosetta) (2004)	Rosetta	MC, Simulated Annealing	MC energy minimization of all-atom Rosetta scoring function	Gap closure term in energy function [48]
Mandell et al. [49] (2009) (Rosetta-KC)	Kinematic closure [49]
Jacobson et al. [34] (2004)	OPLS-AA SGB (A hydrophobic term is added later in [37])	Rotamer Library Buildup	PLOP (Truncated Newton Local Optimization)	Meet in the middle
Zhu et al. [37] (2006)
Zhao et al. [79] (2011)
(PLOP)
Spassov et al. [32] (2008) (Looper)	CHARMM with polar hydrogen force field parameters	Sampling backbone torsion angles in low energy basins of iso-energy contour	Newton-Raphson Minimization	Meet in the middle
Soto et al. [60] (2008) (Loop Builder)	DFIRE (coarse)	Random sampling (same as LOOPY)	PLOP	Direct tweak
OPLS/SBG-NP (fine)
Cui et al. [28] (2008)	Grid-based force field	Local move MC, Simulated Annealing	Steepest Descent Energy Minimization	Filter local moves with reverse proximity criterion
Jamroz and Kolinski [74] (2010)	-	Hybrid Modeller, Rosetta, and CABS	-	
Lee et al. [75] (2010)	DFIRE	Fragment Assembly	Side Chain Optimization	Analytical loop closure
Li et al. [72] (2011) (POS)	Rosetta, DFIRE, Triplet	MOMCMC	PLOP	CCD
Liang et al. [64] (2012) (OSCAR-loop)	Backbone potential, OSCAR force field, OPLS/SGB-NP	Random Buildup	Energy Minimization	CCD

**Table 2:** Loop prediction accuracy in recently published works. The number of loop targets is specified in curly brackets.

Average RMSD (A) {Number of Loop Targets}																		

Methods	Data Source	Loop Length																
		2	3	4	5	6	7	8	9	10	11	12	13	14	15	16	17	18 +
Fiser et al. [47] (2000) (Modeller)	Figure 9	0.5	0.5	1	1	2	2	2.5	3.5	3.5	5.5	6	6.5	6	-	-	-	-
		{40}	{40}	{40}	{40}	{40}	{40}	{40}	{40}	{40}	{40}	{40}	{40}	{40}				
Deane and Blundell [76] (2001) (CODA)	Table V	-	0.78	1.09	1.96	2.36	3.29	3.5	-	-	-	-	-	-	-	-	-	-
			{153}	{184}	{162}	{97}	{78}	{60}										
Xiang et al. [51] (2002) (LOOPY)	Table I	-	-	-	0.85	0.92	1.23	1.45	2.68	2.21	3.52	3.42	-	-	-	-	-	-
					{161}	{107}	{74}	{61}	{58}	{34}	{37}	{21}						
De Bakker et al. [30] (2003) (RAPPER)	Table III	0.35	0.37	0.47	0.9	0.95	1.37	2.28	2.41	3.48	4.94	4.99	-	-	-	-	-	-
		{34}	{34}	{35}	{35}	{36}	{38}	{32}	{37}	{37}	{33}	{34}						
Rohl et al. [48] (Rosetta) (2004)	Table II and Table VI	-	-	0.69	-	-	-	1.45	-	-	-	3.62	5.15					
				{40}				{40}				{40}	{avg. over 10 13- to 35-residue loops}					
Jacobson et al. [34] (2004) (PLOP)	Table IX	-	-	0.2	0.24	0.28	0.3	0.44	0.51	1.09	1.87	1.93	-	-	-	-	-	-
				{35}	{117}	{100}	{82}	{66}	{57}	{40}	{18}	{10}						
Zhu et al. [37] (2006) (PLOP)	Table II	-	-	-	-	-	-	-	-	-	1	1.15	1.25	-	-	-	-	-
												{38}	{31}						{35}
Spassov et al. [32] (2008) (Looper)	Table I	0.26	0.31	0.42	0.49	0.81	1.07	1.33	1.63	2.66	3.35	4.08	-	-	-	-	-	-
		{40}	{40}	{40}	{40}	{40}	{40}	{40}	{40}	{40}	{40}	{40}						
Soto et al. [60] (2008) (Loop Builder)	Table V	-	-	-	-	-	-	1.31	1.88	1.93	2.5	2.65	3.74	-	-	-	-	-
								{63}	{56}	{40}	{54}	{40}	{40}					
Cui et al. [28] (2008)	Table I	-	-	0.75						-	-	-	-	-	-	-	-	-
				{avg. over 14 4- to 9-residue loops}														
Mandell et al. [49] (2009) (Rosetta-KC)	Figure 2	-	-	-	-	-	-	-	-	-	-	0.8	-	-	-	-	-	-
												{63}						
Jamroz and Kolinski [74] (2010)	Table II	-	-	1.07			2.23						-	-	-	7.87		
				{49}			{64}									{73}		
Lee et al. [75] (2010)	Table IV	-	-	0.54	0.92	1.36	1.17	1.87	2.08	3.09	3.43	3.84	-	-	-	-	-	-
				{35}	{35}	{36}	{38}	{32}	{37}	{37}	{33}	{34}						
Li et al. [72] (2011) (POS)	Tables II and III	-	-	0.33			0.58			0.86			0.63	-	-	-	-	-
				{252}			{205}			{68}			{35}					
Zhao et al. [79] (2011) (PLOP)	Table III	-	-	-	-	-	-	-	-	-	-	-	-	1.19	1.55	1.43	2.3	-
														{36}	{30}	{14}	{9}	
Liang et al. [64] (2012) (OSCAR-loop)	Table III	-	-	0.4	0.52	0.7	0.83	1.1	1.6	2.08	2.73	3.58	-	-	-	-	-	-
				{2809}	{1863}	{1456}	{1053}	{862}	{634}	{528}	{392}	{325}						

It is also important to notice that Table 2 does not serve the purpose of comparing prediction accuracy between different methods. First of all, the prediction accuracies in different methods are reported on different loop targets. Some loop targets are significantly “harder” than the others due to strong external influences from ions, ligands, disulfide bonds, and/or interactions with external chains or other units in the crystallographic unit cell. Several difficult loop targets (1poa(79:83), 1eok(A147:A159), 1hxh(A87:A99), and 1qqp(2_161:2_173)) are analyzed in [62]. Secondly, different criteria have been used to measure the accuracies of their prediction results in different methods. The RMSD calculations may be adopted very differently – either based on all heavy atoms, backbone atoms, or Cα atoms only. Moreover, the RMSD comparison may be directly carried out between the predicted model and the native structure, between the model and the relaxed native structure minimized by a force field, or between structures after global superimposition. Thirdly, loops are modeled under different assumptions in different methods. For example, Rosetta repacks all side chains of the protein [48, 49] while most of the other methods keep the native side chain conformations in the rest of the protein during the loop modeling process. Therefore, Table 2 does not form a fair base for comparing performance among different loop prediction methods, but is instead used to reflect the recent progress in loop modeling.

## Summary

Loops play a critical role in performing important biological functions of proteins. However, due to their high flexibility and variability, modeling the 3D structures of loops is more difficult than other secondary structures. Loop structure modeling is regarded as a “mini protein folding problem” under geometric constraints such as loop closure and steric clashes. The computational loop modeling methods can be categorized into template-based and template-free methods. The template-based methods rely on database search, which is limited by the number of known structures in PDB, particularly when modeling relatively long loops. In comparison, the template-free methods can avoid this problem by diversely sampling loop conformation space to search for appropriate structures. Hence, sampling loop conformation space is the cornerstone of the template-free methods. Successful sampling methods rely on accurate and sensitive energy functions, fast buildup mechanism to generate reasonable loop models satisfying geometric constraints, and efficient sampling algorithms.

There has been remarkable advancement in template-free loop structure modeling in the past decade, mainly due to new computational methods as well as increasing number of known structures available in PDB. Quite a few loop modeling methods with various strategies have successfully predicted short loops (< 8 residues) with resolution comparable to experimental results. Several recent methods have even achieved near sub-angstrom accuracy in longer loops up to 13 residues. However, modeling very long loops over 18 residues is a challenge remaining unaccomplished. Recent study by Raval et al. [87] on protein structure refinement using very long (>100µs) MD simulations has shown that inaccuracy in current force fields limits MD-based protein structure refinement. Similarly, given loop modeling as a “mini protein folding problem,” for difficult or long loop targets, while sampling is no longer a critical issue [87], development of more precise energy functions is now the key.
